# One protein, different cell fate: the differential outcome of depleting GRP75 during oxidative stress in neurons

**DOI:** 10.1038/s41419-017-0148-7

**Published:** 2018-01-18

**Authors:** Birgit Honrath, Carsten Culmsee, Amalia M Dolga

**Affiliations:** 1 Institute of Pharmacology and Clinical Pharmacy, University of Marburg, 35043 Marburg, Germany; 2 Department of Molecular Pharmacology, Faculty of Science and Engineering, University of Groningen, 9713 AV Groningen, The Netherlands

The interconnection between the endoplasmic reticulum (ER) and mitochondria to transfer Ca^2+^ into the mitochondrial matrix constitutes a major part of intracellular Ca^2+^ signaling. By increasing mitochondrial Ca^2+^ ([Ca^2+^]_m_) uptake, ER-mitochondrial crosstalk enhances energy production through accelerating mitochondrial respiration, thereby supporting cellular function and survival.

ER-mitochondrial associations are established by multiprotein complexes formed, for instance, by ER-bound inositol-1,4,5-trisphosphate receptor (IP_3_R), mitochondria-resident voltage-dependent anion channel 1 (VDAC1) and the heat shock protein glucose-regulated protein 75 (GRP75)^1^. While IP_3_R and VDAC1 are Ca^2+^-permeable ion channels driving Ca^2+^ flux, GRP75 is essential to maintain the physical contact between the organelles, thereby facilitating the propagation of the Ca^2+^ signal into the mitochondria^2^. Reduced GRP75 expression in tumor cells derived from bone, breast or colon has been linked to an increased susceptibility to cell death, and small molecule GRP75-inhibitory drugs are exploited as a potential therapeutic intervention^3^. However, the relevance of GRP75 for ER-mitochondrial crosstalk in neurons or brain-derived tumor cells is largely unknown.

Under physiological conditions, GRP75 inhibition seemed to activate mitochondrial stress responses such as the mitochondrial unfolded protein response or autophagy in human neuroblastoma SH-SY5Y cells^4^. In contrast, under pathological conditions, GRP75 expression exerted different effects. For instance, in SH-SY5Y cells increased GRP75 expression prevented mitochondrial dysfunction and cell death following proteolytic stress induced by overexpression of mitochondrial ornithine transcarbamylase^5^. In contrast, in human dopaminergic neurons, GRP75 overexpression potentiated the cytotoxic effects of the mitochondrial complex I inhibitor rotenone^6^.

Our recent study published in *Cell Death & Discovery*^7^ provided further insights into the role of GRP75 on mitochondrial dysfunction and cell death in a neuronal model of oxidative stress. Using immortalized hippocampal HT22 cells, we investigated the consequences of GRP75 expression on cell survival in a model of glutamate-induced oxytosis. In this study, inhibition and/or gene silencing of GRP75 *via* siRNA or CRISPR/Cas9-knockout fully prevented the oxidative cell death. In particular, GRP75 depletion exerted protection by preserving the mitochondrial network, preventing mitochondrial membrane depolarization and restoring the mitochondrial redox balance. Furthermore, GRP75 depletion restored Ca^2+^ homeostasis by preventing [Ca^2+^]_m_ overload and late-stage cytosolic Ca^2+^ dysregulation mediated by Ca^2+^ release-activated calcium channel protein 1 (ORAI1). In turn, elevating GRP75 expression increased the sensitivity of neural HT22 cells towards the glutamate challenge. Notably, neither pharmacological inhibition alone nor genetic downregulation of GRP75 had any effect on cell survival or mitochondrial function in control conditions. GRP75 gene silencing neither impaired cell proliferation, nor altered the mitochondrial network, mitochondrial ROS, or the mitochondrial membrane potential. We therefore suggest that the GRP75-dependent ER-mitochondrial coupling is a major determinant of cell fate in conditions of oxidative stress, without affecting mitochondrial function and cell viability in physiological conditions.

GRP75 is a chaperone interacting with other proteins to regulate mitochondrial protein import, and to activate pro-survival signaling pathways through MAPK activation or pro-apoptotic p53-dependent signaling cascades^8, 9^. In hepatocellular carcinoma cells, GRP75 inhibition substantially enhanced cell death which was linked to an increase in the nucleo-cytoplasmic shuttling of the tumor suppressor p53, thereby driving apoptosis^10^. However, p53 function was dispensable for glutamate toxicity in neural HT22 cells^11^, supporting the concept that the primary function of GRP75 was determining ER-mitochondrial contact formation in these cells.

GRP75 is an adapter protein that bridges the ER to mitochondrial membranes through facilitating the interaction between IP_3_R and VDAC1. Using *in situ* proximity ligation assays, we confirmed a pivotal role for GRP75 in physically bridging ER and mitochondrial since pharmacological inhibition significantly reduced the number of ER-mitochondrial interaction sites. Exposing HT22 cells to glutamate induced oxidative cell death which is associated with mitochondrial dysfunction^12^ but without affecting ER function. In these cells, GRP75 knockdown fully blocked cell death by reducing ER-mitochondrial coupling, suggesting that Ca^2+^ signaling along the ER-mitochondrial interface contributed to glutamate toxicity. In some cell types, ER dysfunction can result in impaired mitochondrial function^13^ through increased ER-mitochondrial Ca^2+^ transfer and alterations in mitochondrial respiration. However, ER stress induced in HT22 cells led to caspase-dependent cell death that was independent of mitochondrial damage^14^. In line with these earlier findings, GRP75 inhibition/depletion was not able to rescue ER stress-induced cell death in our study. In addition, mitochondrial damage elicited by the mitochondrial complex I inhibitor rotenone was also not blocked by GRP75 inhibition/downregulation. Thus, the GRP75-mediated regulation of ER-mitochondrial connectivity determines neurotoxicity in paradigms of oxidative stress upstream of mitochondrial damage; yet detrimental ER dysfunction and associated death signaling is apparently independent of GRP75-mediated connectivity to mitochondria.

**Fig. 1:**
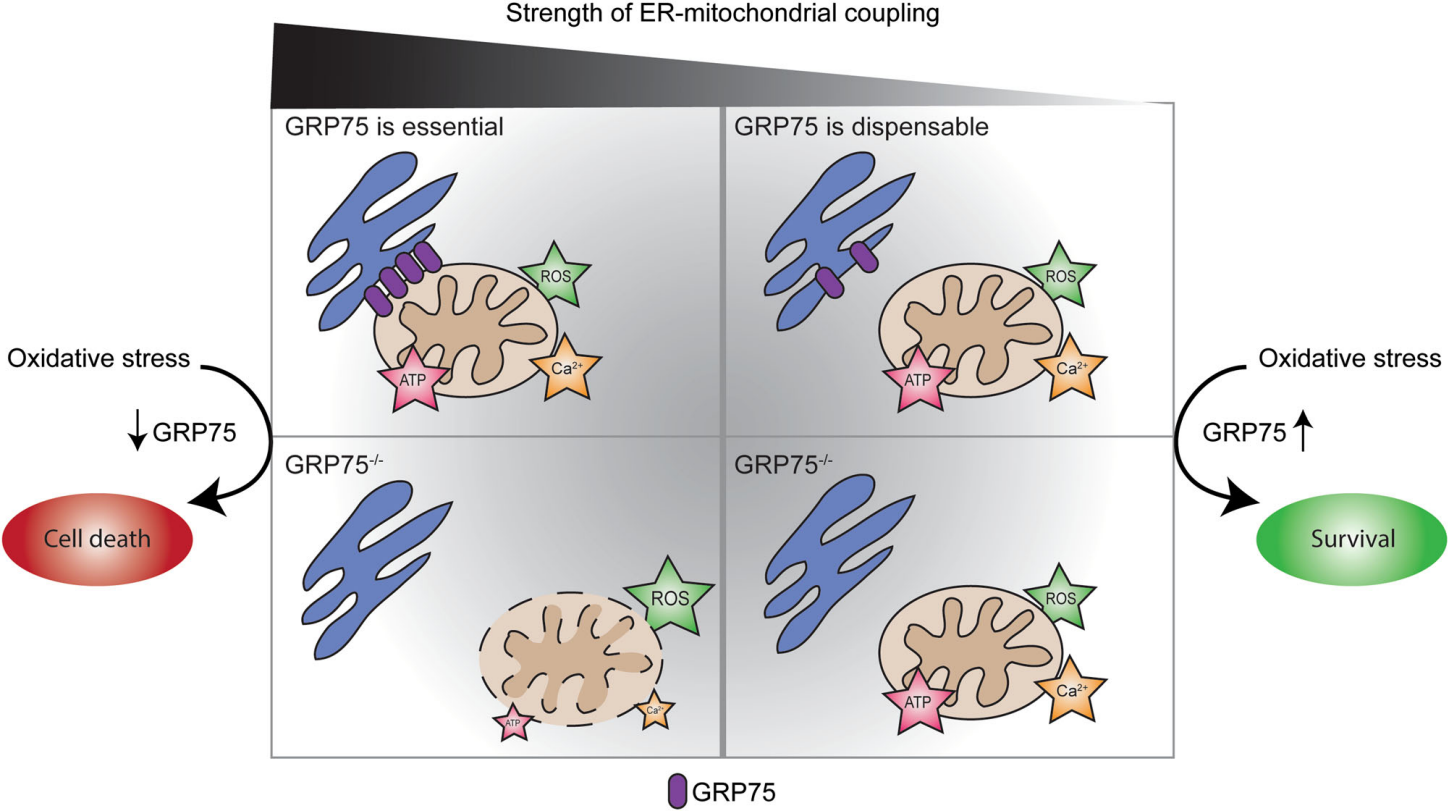
Two outcomes of GRP75 depletion during oxidative stress In brain-derived tumor cells where ER and mitochondria are tightly coupled, GRP75 function at the ER-mitochondrial interface is essential for mitochondrial function and cell viability. Thus, knockdown of GRP75 will reduce ER-mitochondrial coupling, thereby causing mitochondrial dysfunction, ATP depletion and eventually cell death (left panels). In these cells, exposure to oxidative stress may result in a downregulation of GRP75, thereby accelerating mitochondrial damage and cell death. In contrast, in non-cancerous neuronal cells where ER-mitochondrial connectivity is weak, GRP75 function at the ER-mitochondrial interface is dispensable. Hence, knockdown of GRP75 has no harmful consequences for the cells in physiological conditions. Eliciting oxidative stress leads to mitochondrial dysfunction, likely accelerated through an enhanced expression of GRP75 that induced the formation of ER-mitochondrial contact points and accelerated pathological [Ca^2+^]_m_ overload. In this cell type, GRP75 silencing reduced such pathological signaling along the ER-mitochondrial interface in conditions of oxidative stress, thereby preserving mitochondrial function and cell survival

[Ca^2+^]_m_ homeostasis and mitochondrial energy production are regulated by the transfer of small Ca^2+^ microdomains through the ER-mitochondrial interface. Yet large Ca^2+^ microdomains shuttled into mitochondria exceed the capacity of mitochondria to buffer changes in Ca^2+^ concentrations, leading to [Ca^2+^]_m_ overload and to an impairment of mitochondrial function^15^. We show that decreasing GRP75 expression in physiological conditions did not affect mitochondrial function or cell survival while the overexpression of GRP75 increased ER-mitochondrial coupling and accelerated the susceptibility of HT22 cells to oxidative stress. These findings suggest that in neural HT22 cells, ER-mitochondrial coupling mediated by GRP75 is particularly relevant during cellular stress. Furthermore, our results indicate that basal ER-mitochondrial coupling may be low in physiological conditions, and therefore, modulating the coupling strength by GRP75 downregulating is without consequences for cellular function and survival. Thus, we propose different scenarios for GRP75 function depending largely on the cell type and signaling context (Fig. 1). In the first scenario representing published findings in brain-derived tumor cells, ER and mitochondria are tightly associated which is essential for mitochondrial integrity and function. Therefore, GRP75 knockdown was accompanied by disruption of ER-mitochondrial connectivity, mitochondrial dysfunction and cell death. In these cell types, oxidative stress disrupted GRP75-dependent ER-mitochondrial connectivity, thus restoring GRP75 expression then prevented mitochondrial dysfunction and cell death. In the second, alternative scenario representing neuronal cells, ER-mitochondrial connections are dispensable and, therefore, the knockdown of GRP75 has no effect on mitochondrial function in physiological conditions, as we have demonstrated in neural HT22 cells in our study. Exposing these cells to oxidative stress, however, may potentiate Ca^2+^ transfer and associated signaling along the ER-mitochondrial axis, likely attributed to increased GRP75 expression, thereby amplifying the cytotoxic effects. Hence, as reported in our current study, decreasing ER-mitochondrial coupling through depletion of GRP75 renders resistance against oxidative cell death in neuronal cells.
